# Nicotinamide Riboside Augments the Aged Human Skeletal Muscle NAD^+^ Metabolome and Induces Transcriptomic and Anti-inflammatory Signatures

**DOI:** 10.1016/j.celrep.2019.07.043

**Published:** 2019-08-13

**Authors:** Yasir S. Elhassan, Katarina Kluckova, Rachel S. Fletcher, Mark S. Schmidt, Antje Garten, Craig L. Doig, David M. Cartwright, Lucy Oakey, Claire V. Burley, Ned Jenkinson, Martin Wilson, Samuel J.E. Lucas, Ildem Akerman, Alex Seabright, Yu-Chiang Lai, Daniel A. Tennant, Peter Nightingale, Gareth A. Wallis, Konstantinos N. Manolopoulos, Charles Brenner, Andrew Philp, Gareth G. Lavery

**Affiliations:** 1 Institute of Metabolism and Systems Research, University of Birmingham, Birmingham, UK; 2 Centre for Endocrinology, Diabetes and Metabolism, Birmingham Health Partners, Birmingham, UK; 3 MRC-Arthritis Research UK Centre for Musculoskeletal Ageing Research, Institute of Inflammation and Ageing, University of Birmingham, Birmingham, UK; 4 Department of Biochemistry, Carver College of Medicine, University of Iowa, Iowa City, IA, USA; 5 School of Sport, Exercise and Rehabilitation Sciences, University of Birmingham, Birmingham, UK; 6 Centre for Human Brain Health, University of Birmingham, Birmingham, UK; 7 School of Psychology, University of Birmingham, Birmingham, UK; 8 Institute of Translational Medicine, University Hospitals Birmingham NHS Foundation Trust, Birmingham, UK; 9 Diabetes and Metabolism Division, Garvan Institute of Medical Research, Sydney, NSW, Australia; 10 Faculty of Medicine, St. Vincent’s Clinical School, Sydney, UNSW, Australia

**Keywords:** nicotinamide adenine dinucleotide, metabolism, aging, inflammation, cell adhesion

## Abstract

Nicotinamide adenine dinucleotide (NAD^+^) is modulated by conditions of metabolic stress and has been reported to decline with aging in preclinical models, but human data are sparse. Nicotinamide riboside (NR) supplementation ameliorates metabolic dysfunction in rodents. We aimed to establish whether oral NR supplementation in aged participants can increase the skeletal muscle NAD^+^ metabolome and if it can alter muscle mitochondrial bioenergetics. We supplemented 12 aged men with 1 g NR per day for 21 days in a placebo-controlled, randomized, double-blind, crossover trial. Targeted metabolomics showed that NR elevated the muscle NAD^+^ metabolome, evident by increased nicotinic acid adenine dinucleotide and nicotinamide clearance products. Muscle RNA sequencing revealed NR-mediated downregulation of energy metabolism and mitochondria pathways, without altering mitochondrial bioenergetics. NR also depressed levels of circulating inflammatory cytokines. Our data establish that oral NR is available to aged human muscle and identify anti-inflammatory effects of NR.

## Highlights

• NR supplementation in aged subjects augments the skeletal muscle NAD^+^ metabolome
• NR supplementation does not affect skeletal muscle mitochondrial bioenergetics
• NR supplementation reduces levels of circulating inflammatory cytokines

## Graphical Abstract

**Figure:**
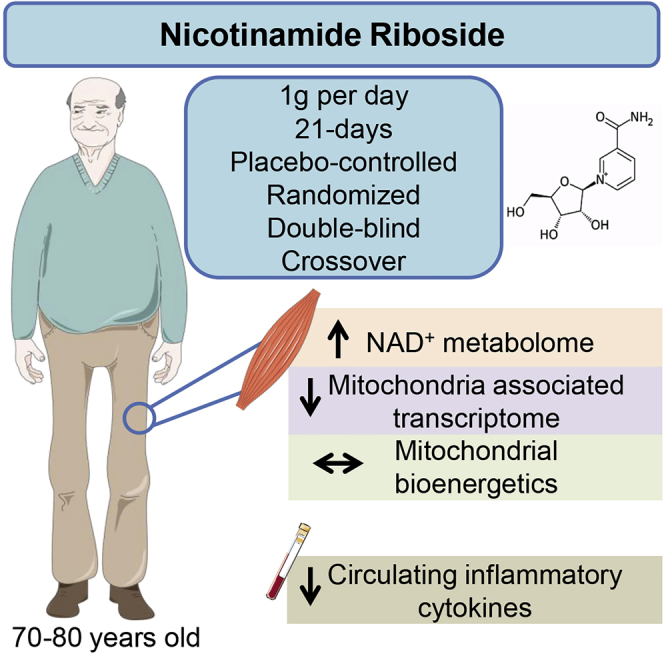

## Teaser

Elhassan et al. show that oral nicotinamide riboside increases the NAD^+^ metabolome in aged human skeletal muscle, without apparently altering mitochondrial bioenergetics. Measures of muscle and whole-body metabolism are also unchanged. Nicotinamide riboside reduces the levels of circulating inflammatory cytokines. Studies in relevant human disease models are warranted.

## Introduction

Aging is characterized by a decline in metabolic and physiological functions of all organs within the body. A hallmark feature of aging is the progressive loss of skeletal muscle mass and function that can progress to sarcopenia, which is associated with significant morbidity and mortality and substantial healthcare costs (Kim and Choi, 2013, Sousa et al., 2016). Exercise is considered a frontline modality to combat age-related muscle decline (Costford et al., 2010). However, nutritional strategies may also offer an effective countermeasure to age-associated morbidities and promote healthy muscle aging (Bogan and Brenner, 2008).

Nicotinamide adenine dinucleotide (NAD^+^) homeostasis is critical to cell and organismal function. In addition to its classical role in redox metabolism, NAD^+^ is a substrate for enzymes such as sirtuins, poly-ADPribose polymerases (PARPs), and cyclic ADPribose synthetases that regulate key cellular processes of energy metabolism, DNA damage repair, and calcium signaling (Yoshino et al., 2018). Improving NAD^+^ availability via the supplementation of the NAD^+^ precursor nicotinamide riboside (NR) (Bieganowski and Brenner, 2004, Trammell et al., 2016a) has emerged as a potential strategy to augment tissue-specific NAD^+^ homeostasis and improve physiological function (Elhassan et al., 2017). A range of physiological stresses associated with the depletion of NAD^+^ and/or nicotinamide adenine dinucleotide phosphate hydrogen (NADPH) have been ameliorated with NR supplementation in mice, including prevention of noise-induced hearing loss (Brown et al., 2014), resistance to weight gain (Cantó et al., 2012), reduction of blood glucose, hepatic steatosis and neuropathy on a high-fat diet (Trammell et al., 2016b), improvement of cardiac function in genetic cardiomyopathy (Diguet et al., 2018), and prevention of cortical neuronal degeneration (Vaur et al., 2017). Depletion of the enzyme nicotinamide phosphoribosyltransferase (NAMPT), rate-limiting for NAD^+^ biosynthesis, in mouse skeletal muscle severely diminishes NAD^+^ levels and induces sarcopenia. Oral repletion of NAD^+^ with NR in this model rescued pathology in skeletal muscle in a cell-autonomous manner (Frederick et al., 2016). However, recent data in mice tracing NAD^+^ fluxes questioned whether oral NR has the ability to access muscle (Liu et al., 2018). Thus, whether oral NR can augment the human skeletal muscle NAD^+^ metabolome is currently unknown.

A decline in NAD^+^ availability and signaling appears to occur as part of the aging process in many species (Gomes et al., 2013, Mouchiroud et al., 2013), though there is a paucity of data to confirm that this is the case in human aging. NR and nicotinamide mononucleotide (NMN) are reported to extend life spans (Zhang et al., 2016) and enhance metabolism in aged mice (Mills et al., 2016). To date, NR supplementation studies in humans have focused on cardiovascular (Martens et al., 2018), systemic metabolic (Dollerup et al., 2018), exercise (Dolopikou et al., 2019), and safety (Conze et al., 2019) end-points, but have not addressed advanced aging, tissue metabolomic changes, or effects on muscle metabolism and function.

Herein, we set out to study if oral NR is available to aged human skeletal muscle and whether potential effects on muscle metabolism can be detected. We conducted a 21-day NR supplementation intervention in a cohort of 70–80-year-old men in a placebo-controlled, double-blind, crossover trial. We demonstrate that NR augments the skeletal muscle NAD^+^ metabolome, inducing a gene expression signature suggestive of downregulation of energy metabolism pathways, but without affecting muscle mitochondrial bioenergetics or metabolism. Additionally, we show that NR suppresses specific circulating inflammatory cytokine levels.

## Results

### Oral NR Is Safe and Well-Tolerated in Aged Adults

Twelve aged (median age of 75 years) and marginally overweight (median BMI of 26.6 kg/m^2^; range 21–30), but otherwise healthy, men were recruited and orally supplemented with 1-g NR per day for 21 days in a placebo-controlled, randomized, double-blind, crossover design, with 21 days’ washout period between phases. Baseline characteristics of participants are included in Table S1. NR chloride (Niagen) and a placebo were provided as 250-mg capsules (ChromaDex), and subjects were instructed to take two in the morning and two in the evening. All participants completed the study visits (5 in total) and assessments according to protocol (Figure S1). Visit 1 was a screening and enrollment visit, while visit 4 was after the washout period, and only fasting blood and 24-h urine were collected. The protocol design for visits 2, 3, and 5 included muscle biopsy, fasting blood analyses, glucose tolerance test, muscle arterio-venous difference technique, venous occlusive plethysmography, and indirect calorimetry analysis (Figure S1). NR was well tolerated, and screening for a range of hematological and clinical biochemistry safety parameters (including renal, liver, and thyroid functions) revealed no adverse effects (Table S2). No clinical adverse events were reported during the intervention in either phase. Of note, four participants (33.3%), blinded to the intervention arm, self-reported a noticeable increase in libido while on NR. There were no such reports while on the placebo.

### Oral NR Augments the Skeletal Muscle NAD^+^ Metabolome

**Figure 1:**
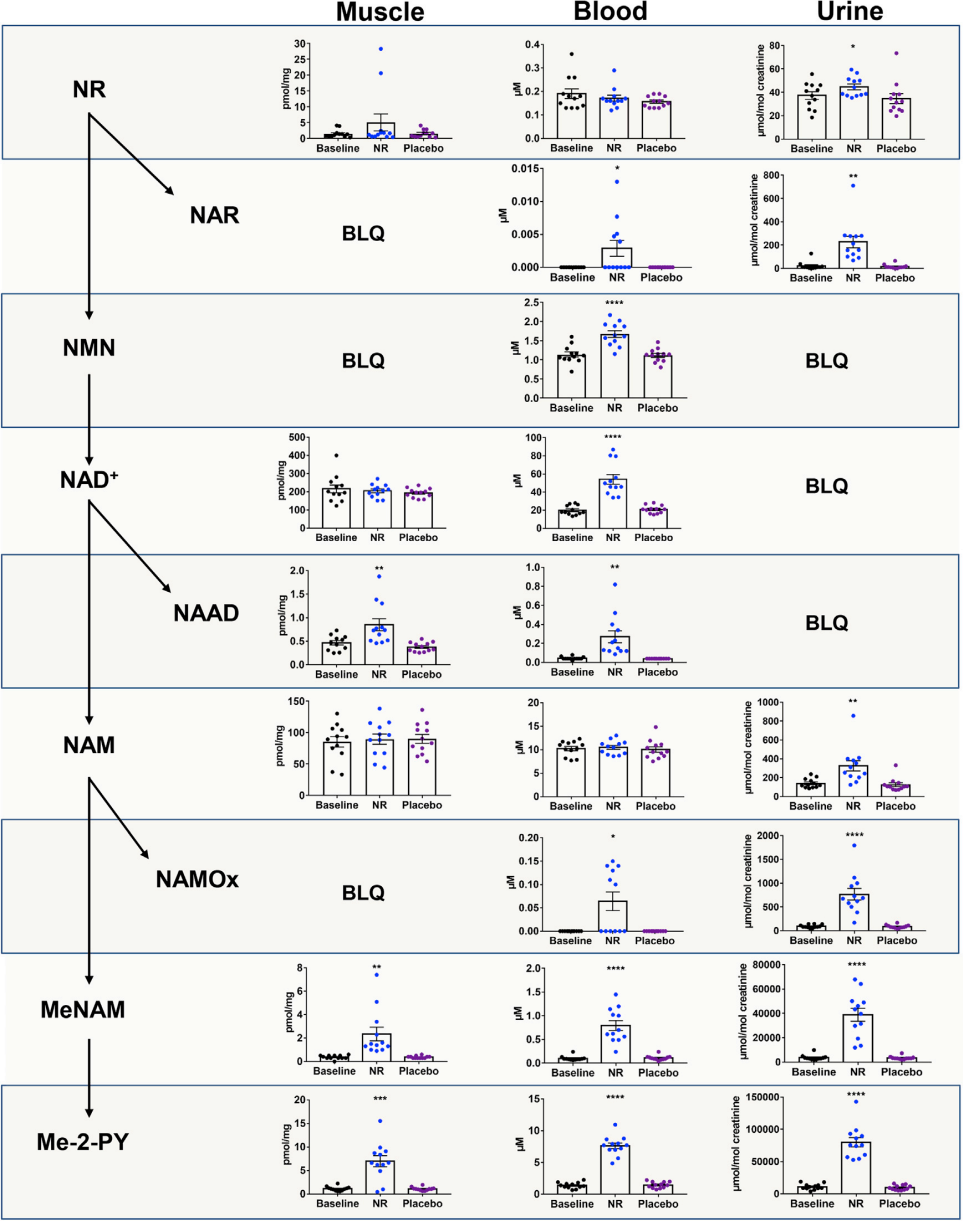
NR Augments the Human Skeletal Muscle NAD^+^ Metabolome Schematic representation of nicotinamide riboside (NR) metabolism within the nicotinamide adenine dinucleotide (NAD^+^) metabolome, accompanied by observed levels of metabolites measured using LC-MS/MS in skeletal muscle, whole blood, and urine at baseline and after each of the NR and placebo periods. NAD^+^ metabolomics data at the end of the washout period are shown in Table S3. Skeletal muscle data were normalized to the weight of the muscle pellet used for extraction. Urine data were normalized to urinary creatinine. Other metabolites are shown in Figure S2. Data are obtained from 12 participants at each phase and presented as mean ± SEM. Significance was set at p < 0.05 using paired t tests and represents the differences between NR and the placebo and between NR and baseline. The absence of significance symbols indicates a lack of statistical significance. BLQ, below limit of quantification; NMN, nicotinamide mononucleotide; NAAD, nicotinic acid adenine dinucleotide; NAM, nicotinamide; NAMOx, nicotinamide N-oxide; MeNAM, N-methyl nicotinamide; Me-2-py, N1-Methyl-2-pyridone-5- carboxamide.

To assess the effects of NR supplementation on NAD^+^ metabolism, we used a targeted liquid chromatography-mass spectrometry (LC-MS/MS) method (Trammell and Brenner, 2013) to quantify the NAD^+^ metabolome in skeletal muscle, whole venous blood, and urine. We examined the NAD^+^ metabolome in skeletal muscle biopsies from all participants in a fasted state at baseline and after the NR and placebo phases, 14 h after the last dose and prior to the physiological assessments. Samples were collected 14 h after the last dose so participants could attend in a fasted state, as well as to evaluate the effects of longer-term NR administration rather than those of an acute dose. Fourteen metabolites were measured in muscle extracts (Figures 1 and S2A; Table S3). NR was detectable in muscle but was not elevated in the NR supplementation period (NR 1.4 pmol/mg μM versus placebo 1.25 pmol/mg; p = 0.23). Consistent with nicotinic acid adenine dinucleotide (NAAD) as a highly sensitive biomarker of NR supplementation and an enhanced rate of NAD^+^ synthesis (Trammell et al., 2016a), we found that oral NR resulted in a 2-fold increase in muscle NAAD (NR 0.73 pmol/mg versus placebo 0.35 pmol/mg; p = 0.004), without an increase in NAD^+^ (NR 210 pmol/mg versus 197 pmol/mg; p = 0.22). NR supplementation did not affect muscle nicotinamide (NAM) (NR 92.0 pmol/mg versus placebo 86.5 pmol/mng; p = 0.96). However, remarkably, we detected 5-fold increases in the products of NAM methylation clearance pathways; N-methyl nicotinamide (MeNAM; NR 1.45 pmol/mg versus placebo 0.35 pmol/mg; p = 0.006), N1-methyl-2-pyridone-5-carboxamide (Me-2-py; NR 6.6 pmol/mg versus placebo 1.1 pmol/mg; p < 0.001), and N1-methyl-4-pyridone-5-carboxamide (Me-4-py; NR 1.6 pmol/mg versus placebo 0.3 pmol/mg; p < 0.001) (Figures 1 and S2A; Table S3).

In the blood, we measured 15 metabolites from each participant at baseline and following each of the NR, placebo, and washout periods (Figures 1 and S2B; Table S3). NR was also detectable in the blood but was not increased, compared to the placebo at 14 h after the last dose of NR (NR 0.16 μM versus placebo 0.15 μM; p = 0.31). This is expected, as the predicated Cmax for NR is approximately 3 h (Airhart et al., 2017). NR increased the concentrations of NAD^+^ >2-fold (NR 47.75 μM versus placebo 20.90 μM; p < 0.001) and NMN 1.4-fold (NR 1.63 μM versus placebo 1.13 μM; p < 0.001). A recent study reported that oral NR is rapidly metabolized in the liver to NAM, which can enhance tissue NAD^+^ metabolomes (Liu et al., 2018). However, chronic NR supplementation did not elevate NAM in the blood (NR 10.60 μM versus placebo 9.50 μM; p = 0.41). Again, NAM urinary clearance pathways were highly active following NR, with marked excess of MeNAM (NR 0.66 μM versus placebo 0.10 μM; p < 0.001), Me-2-py (NR 7.69 μM versus placebo 1.44 μM; p < 0.001), and Me-4-py (NR 3.82 μM versus placebo 0.48 μM; p < 0.001) (Figures 1 and S2B; Table S3). NR elevated blood NAAD levels by 4.5-fold (NR 0.18 μM versus placebo 0.04 μM; p < 0.001).

Urinary NAD^+^ metabolomics showed that NR was detectable and increased with NR supplementation (NR 41.5 μmol/mol creatinine versus placebo 31.7 μmol/mol creatinine; p = 0.02) (Figure 1). Furthermore, a near-20-fold increase in nicotinic acid riboside (NAR; NR-185.5 μmol/mol creatinine versus placebo-10.3 μmol/mol creatinine; p = 0.001) was observed. This observation may support the suggestion that NR supplementation leads to retrograde production of NAAD, nicotinic acid mononucleotide (NAMN), and NAR (Trammell et al., 2016a). However, direct NR transformation into NAR cannot be excluded. Unlike muscle and blood, NAM was elevated in the urine 2.5-fold (NR-282 μmol/mol creatinine versus placebo-106.5 μmol/mol creatinine; p = 0.004). These data establish the extent and breadth of changes to NAD^+^ metabolites in human muscle, blood, and urine after NR supplementation. The data indicate that oral NR greatly boosts the blood NAD^+^ metabolome without an increase in NAM, increases muscle NAD^+^ metabolism, and leads to the disposal of urinary clearance products.

### Oral NR Results in Downregulation of Gene Sets Associated with Energy Metabolism in Skeletal Muscle

**Figure 2:**
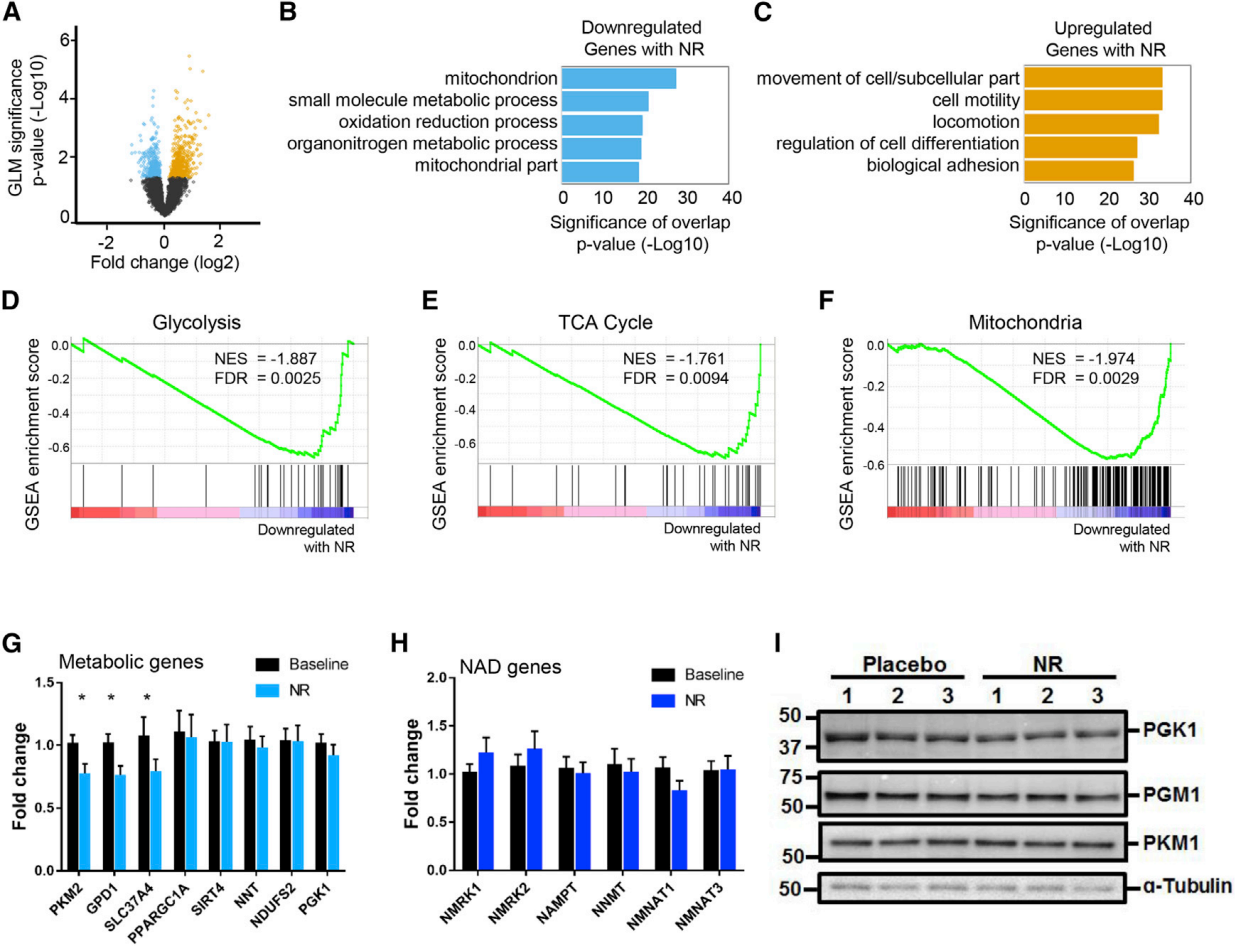
NR Supplementation Induces a Transcriptional Signature in Human Skeletal Muscle (A) Differential gene expression analysis on baseline and NR-treated muscle samples (n = 12 at each phase). Volcano plot of differential gene expression between baseline and NR treated human muscle samples. Fold change (Log2, x axis) of gene expression is plotted against p value for differential gene expression (–Log10, y axis). Colored dots represent Ensembl genes that are either upregulated (in orange) or downregulated (in blue) upon NR supplementation at a p value < 0.05. (B and C) Gene Ontology analysis of significantly dysregulated genes upon NR supplementation for (B) downregulated genes and (C) upregulated genes. Gene Ontology analysis was performed using GSEA. Bars represent the p value (–Log10) of overlap from hypergeometric distribution. (D) Gene set enrichment analysis (GSEA) suggests that genes belonging to the gene set “glycolysis” are downregulated upon NR supplementation. The normalized enrichment score (NES) and nominal p value are presented on the top-left corner of the graph. (E) As in (D), but for genes involved in the TCA cycle. (F) As in (D), but for genes involved in the gene set “mitochondria.” (G) A qPCR analysis of a select panel of downregulated genes identified through differential gene expression analysis. *GAPDH* was used as housekeeping gene. Error bars represent SEM (n = 12). (H) As in (G), but for NAD^+^ pathway-related genes. (I) Quantification of phosphoglycerate kinase 1 (PGK1), phosphoglucomutase 1 (PGM1), and pyruvate kinase M1 (PKM1) proteins using immunoblotting assay. Tubulin was used as a loading control. Data are obtained from 12 participants at each phase and wherever relevant are presented as mean ± SEM. Significance was set at p < 0.05. The absence of significance symbols indicates a lack of statistical significance.

We next assessed NR-mediated transcriptional changes in skeletal muscle. RNA sequencing followed by differential gene expression (DGE) analysis of muscle biopsies from the 12 participants revealed 690 upregulated and 398 downregulated genes between baseline and NR supplementation at p value < 0.05 (Figure 2A; Table S4). Using gene annotation analysis (gene set enrichment analysis [GSEA]) (Mootha et al., 2003, Subramanian et al., 2005), we examined the enrichment of genes that belong to known molecular pathways in our list of up- or downregulated genes. Our results suggest that genes significantly downregulated with NR supplementation were enriched in pathways relating to energy metabolism, including those of glycolysis, tricarboxylic acid (TCA) cycle, and mitochondria (Figure 2B; Table S5). This is consistent with the recent discovery that oral NR depresses mitochondrial membrane potential while improving blood stem cell production in mice (Vannini et al., 2019).

Pathways upregulated upon NR supplementation prominently belonged to Gene Ontology categories such as cell adhesion, actin cytoskeleton organization, and cell motility (Figure 2C). This supports a previously identified role for the NAD^+^-generating enzyme NR kinase 2b (Nrk2b) in zebrafish skeletal muscle cell adhesion (Goody et al., 2010).

We next examined all the genes that belonged to the glycolysis, mitochondrial, and TCA cycle pathways and found that they were predominantly downregulated following NR supplementation, whereas 10 control gene sets of the same size and expression level were not (Figures 2D–2F and S3A). Similarly, we found that the genes belonging to the Gene Ontology terms actin filament-based process, cell motility, and biological cell adhesion were mainly upregulated upon NR supplementation (Figures S3B and S3C).

In agreement with the DGE analysis, quantitative real-time PCR showed downregulation of selected genes involved in energy metabolism (Figure 2G). We found no changes in the transcript levels of key genes involved in NAD^+^ metabolism, corroborating the DGE analysis (Figure 2H). We also verified some of the upregulated targets by qPCR (Figure S3D) and undertook some immunoblotting validation (Figure S3D).

As it has previously been shown that NR increases glycolysis in mouse cardiac cells (Diguet et al., 2018), and because our data do not support an NR-mediated transcriptional upregulation of glycolysis related genes, we examined protein expression levels of glycolytic enzymes in our muscle biopsies and show them to be unchanged after NR (Figure 2I).

### Three Weeks of Oral NR Does Not Alter Skeletal Muscle Mitochondrial Bioenergetics or Hand-Grip Strength

**Figure 3:**
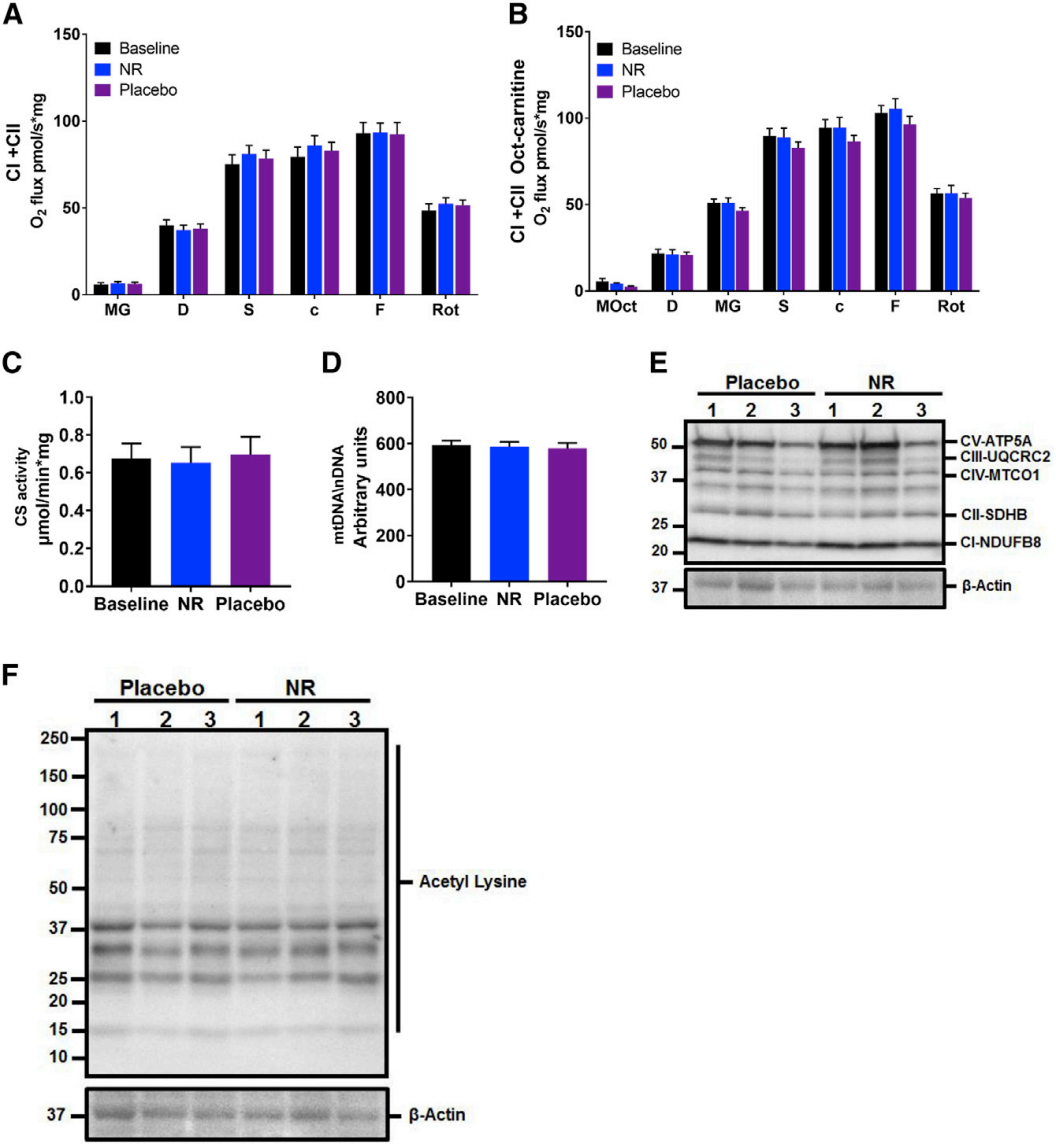
Human Skeletal Muscle Mitochondrial Bioenergetics Remain Unaltered with NR Supplementation (A) Mitochondrial respiration of permeabilized muscle fibers upon the addition of complex I and complex II substrates at baseline and after 3 weeks of supplementation of NR and the placebo. MG, malate and glutamate; D, ADP; S, succinate; c, cytochrome C; F, FCCP; Rot, rotenone. Data are normalized to muscle fiber weight. (B) Mitochondrial respiration as per (A), but with the prior addition of the fatty acid conjugate octanoyl-carnitine to malate (MOct). (C) Citrate synthase (CS) activity in human skeletal muscle at baseline and after NR and the placebo. (D) Relative PCR expression of mitochondrial DNA (mtDNA) to nuclear DNA (nDNA) at baseline and after NR and the placebo, expressed as arbitrary units. (E) Western blot showing the expression of selected mitochondrial proteins in skeletal muscle lysates compared to β-actin as housekeeping protein. (F) Western blot showing the expression of acetylation proteins in skeletal muscle lysates compared to β-actin as housekeeping protein. Data are obtained from 12 participants at each phase and wherever relevant are presented as mean ± SEM. Significance was set at p < 0.05. The absence of significance symbols indicates a lack of statistical significance.

Several preclinical studies suggest that NR enhances mitochondrial energy programs in skeletal muscle (Cantó et al., 2012, Frederick et al., 2016) through mechanisms that involve redox and sirtuins activation. Therefore, we undertook a detailed assessment of muscle mitochondrial respiration in biopsies after NR supplementation using high-resolution respirometry, the gold standard method for the *ex vivo* assessment of mitochondrial function. No differences were detected between the NR and placebo groups in skeletal muscle complex I- and complex II-mediated oxidative phosphorylation and maximal respiratory capacity, with (Figure 3A) and without (Figure 3B) the prior addition of the fatty acid conjugate octanoyl-carnitine. In line with this, the activity of citrate synthase, commonly used as a quantitative measure of mitochondrial content (Larsen et al., 2012) (Figure 3C), and mitochondrial copy number (mtDNA) (Phielix et al., 2008) (Figure 3D) were unchanged by NR supplementation. Similarly, levels of skeletal muscle biopsy mitochondrial resident proteins, directly involved in the electron transport chain, were unaltered upon NR supplementation (Figure 3E). We then tested whether the NR-driven increase in the NAD^+^ metabolome translates into higher sirtuin-mediated deacetylation activity, and we performed western blotting to assess pan-acetylation status, but again did not detect NR-mediated changes to muscle protein acetylation (Figure 3F).

Data from rodents suggest that NAD^+^ supplementation can improve the physiological function in skeletal muscle decline (Cantó et al., 2012, Frederick et al., 2016, Mills et al., 2016); thus, we used hand-grip strength as a surrogate marker for muscle function, but one cannot expect hand-grip strength to change after 3 weeks of NR supplementation and without muscle training. Hand-grip strength correlates with leg strength and is used for the diagnosis of sarcopenia and frailty, and it is a better predictor for clinical outcomes than low muscle mass (Lauretani et al., 2003). A decline in hand-grip strength is observed after the third decade of life (when median peak strength is 51 kg of force in men) (Dodds et al., 2014), dropping to median of 33.8 kg of force in our participants. A grip strength of <30 kg of force in men is a diagnostic criterion for sarcopenia (Cruz-Jentoft et al., 2010, Cruz-Jentoft et al., 2019). After 3 weeks of supplementation, we did not observe any differences in the participants’ peak hand-grip strengths (NR 32.5 kg versus placebo 34.7 kg; p = 0.96) or body-weight-adjusted relative strength (NR 2.4 versus placebo 2.3; p = 0.96) between NR and the placebo (Figure S4).

### Oral NR Does Not Alter Skeletal Muscle Blood Flow or Substrate Utilization

**Figure 4:**
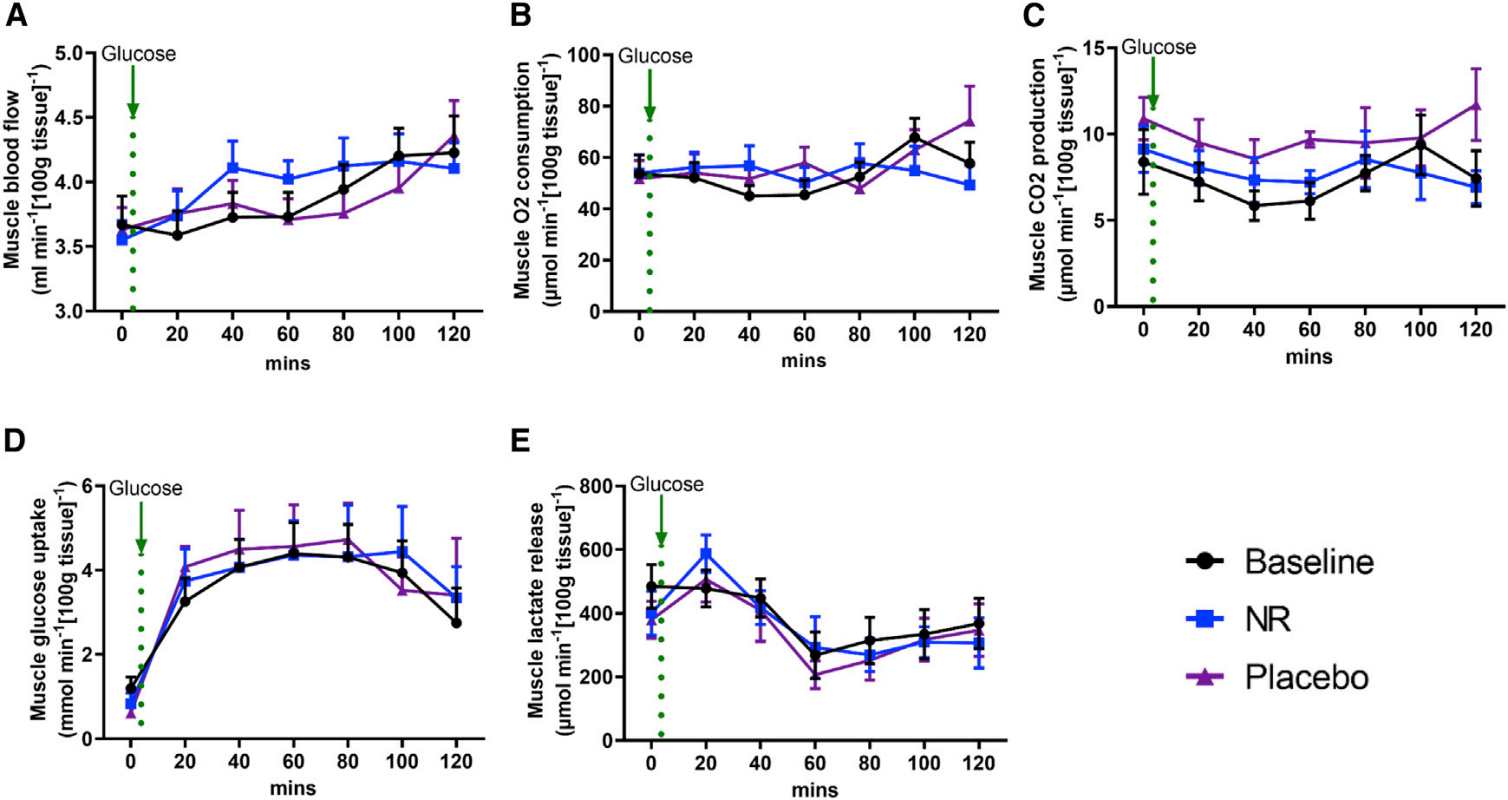
Forearm Muscle Blood Flow and Substrate Utilization Are Unaffected by NR Supplementation (A) Muscle blood flow using venous occlusive plethysmography at baseline and after the NR and placebo phases. The green dotted line represents when 75 g of oral glucose load was taken. (B and C) Muscle O_2_ consumption (B) and CO_2_ production (C) at baseline and after NR and the placebo. The green dotted line represents when 75 g of oral glucose load was taken. (D and E) Muscle glucose uptake (D) and lactate release (E) at baseline and after NR and the placebo. The green dotted line represents when 75 g of oral glucose load was taken. Data are obtained from 12 participants at each phase and presented as mean ± SEM. Significance was set at p < 0.05 using a paired t test. The absence of significance symbols indicates a lack of statistical significance.

Recent mouse data showed that NMN increases angiogenesis and muscle blood flow (Das et al., 2018). Therefore, we used venous occlusive plethysmography to test forearm muscle blood flow in the participants in a non-invasive manner (Greenfield et al., 1963). At fasting, no NR-mediated differences were detected in muscle blood. Following oral glucose load, muscle blood flow gradually increases, but again with no differences between NR and the placebo (Figure 4A).

We then used the arteriovenous difference method (see Method Details) to compare substrate utilization across the forearm muscle (between arterial blood supplying the muscle and venous blood drained from the muscle), with muscle blood flow taken into consideration (Bickerton et al., 2007). No differences were detected in O_2_ consumption (Figure 4B) and CO_2_ production (Figure 4C) between NR and the placebo at the fasting state and in response to oral glucose. Muscle glucose uptake was increased following oral glucose before a gradual decline. No changes were observed in muscle glucose handling with or without NR (Figure 4D). Oral glucose reduced lactate production from muscle, again without a difference in response between NR and the placebo (Figure 4E). These data suggest that the skeletal muscle transcriptomic signature of downregulated mitochondrial and glycolysis genes is undetectable when considered at a functional level.

### Oral NR Does Not Alter Systemic Cardiometabolic Parameters

**Figure 5:**
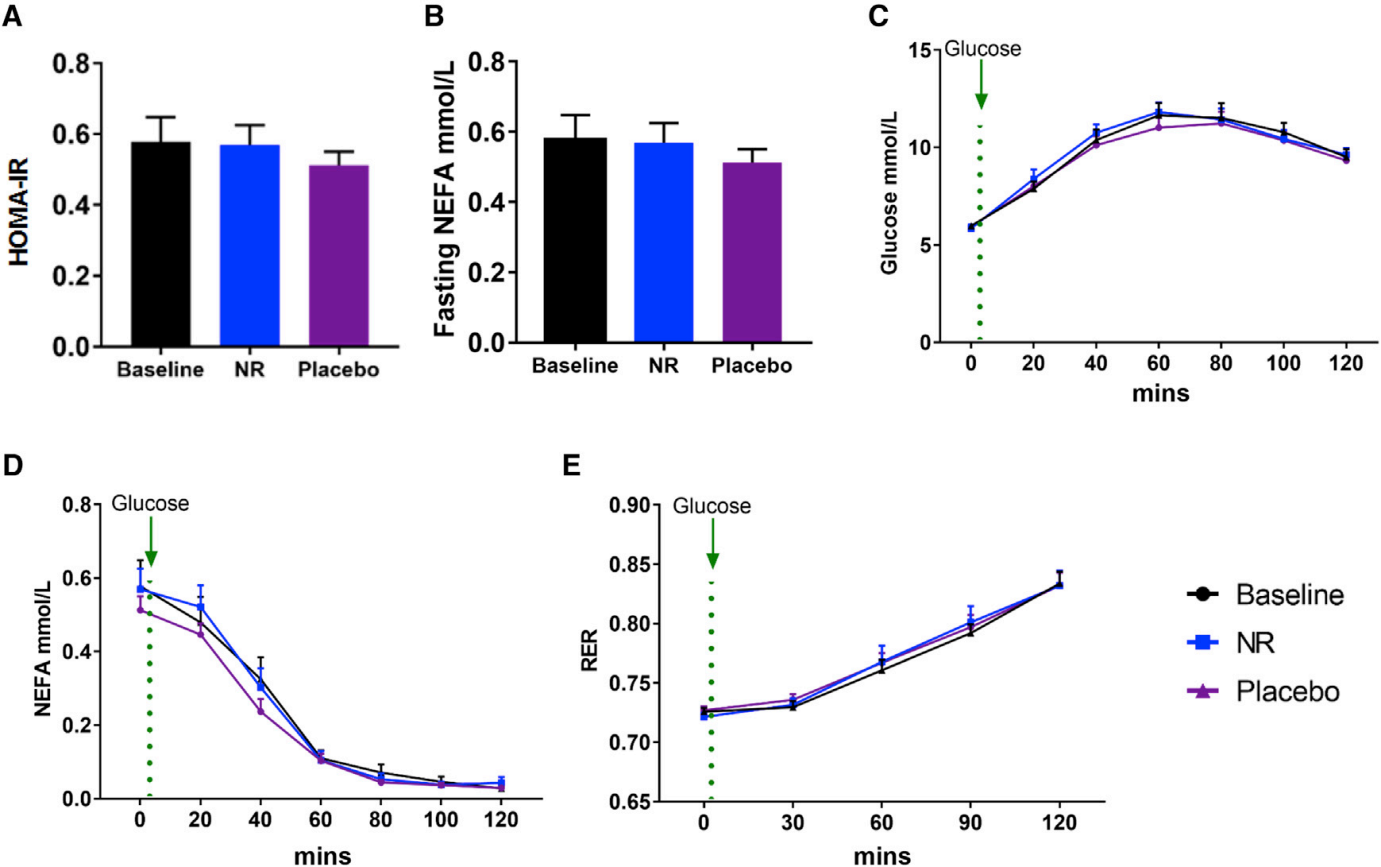
Systemic Readouts of Metabolism Are Unaltered with NR Supplementation (A) HOMA-IR at baseline and after NR and the placebo. (B) Fasting non-esterified fatty acid (NEFA) level at baseline and after NR and the placebo. (C) Plasma glucose response in a glucose tolerance test at baseline and after NR and the placebo. The green dotted line represents when 75 g of oral glucose load was taken. (D) Plasma NEFA response in a glucose tolerance test at baseline and after NR and the placebo. The green dotted line represents when 75 g of oral glucose load was taken. (E) Respiratory exchange ratio (RER) at baseline and after NR and the placebo. The green dotted line indicates when 75 g of oral glucose was taken. Data are obtained from 12 participants at each phase and presented as mean ± SEM. Significance was set at p < 0.05. The absence of significance symbols indicates a lack of statistical significance.

Several preclinical studies have described that NAD^+^ supplementation promotes a resistance to weight gain, ameliorates markers of cardiometabolic risk, and improves metabolic flexibility (Yoshino et al., 2018). As NR increased the circulating levels of the NAD^+^ metabolome, we reasoned that there was increased NAD^+^ availability and turnover in central and peripheral tissues and assessed for resultant cardiometabolic adaptations. Two studies—one of 12 weeks of NR supplementation at 2 g/day in subjects with obesity (Dollerup et al., 2018) and one of 6 weeks of NR supplementation at 1 g/day in older adults (Martens et al., 2018)—suggested potential benefits with respect to fatty liver and blood pressure, respectively. Data for participants at baseline and following NR or the placebo are reported in Table S1. There were no changes in body weight, blood pressure, lipid profile, fasting glucose and insulin (Table S1), and homeostatic model assessment of insulin resistance (HOMA-IR) (Figure 5A). A rebound increase in non-esterified fatty acids (NEFAs) has previously been associated with the nicotinic acid analog, acipimox (van de Weijer et al., 2015); however, NR did not produce this effect in our trial (Figure 5B). Glucose handling was studied using an oral glucose tolerance test, with no effect of NR measured in glucose levels during the 2-h test (Figure 5C). Following the oral glucose load and the consequent insulin stimulation, NEFA levels were appropriately suppressed, and no difference in this response was observed between NR and the placebo (Figure 5D). We also assessed metabolic flexibility using indirect calorimetry to derive respiratory exchange ratios (RERs; calculated as VCO_2_ expired/VO_2_ consumed), reflecting whole-body metabolic substrate use. Measurements were initiated in the fasted state and monitored during the response to the oral glucose load. The median fasting RER was appropriate at 0.72 and 0.73 for the NR and placebo periods, respectively (p = 0.68). In response to glucose, RER values significantly increased, indicating adequate switching from lipids toward carbohydrate utilization, with no differences in response to 3 weeks of NR supplementation observed at 2 h (RERs 0.83 and 0.84 for NR and the placebo, respectively) (Figure 5E).

### Oral NR Depresses Circulating Levels of Inflammatory Cytokines

**Figure 6:**
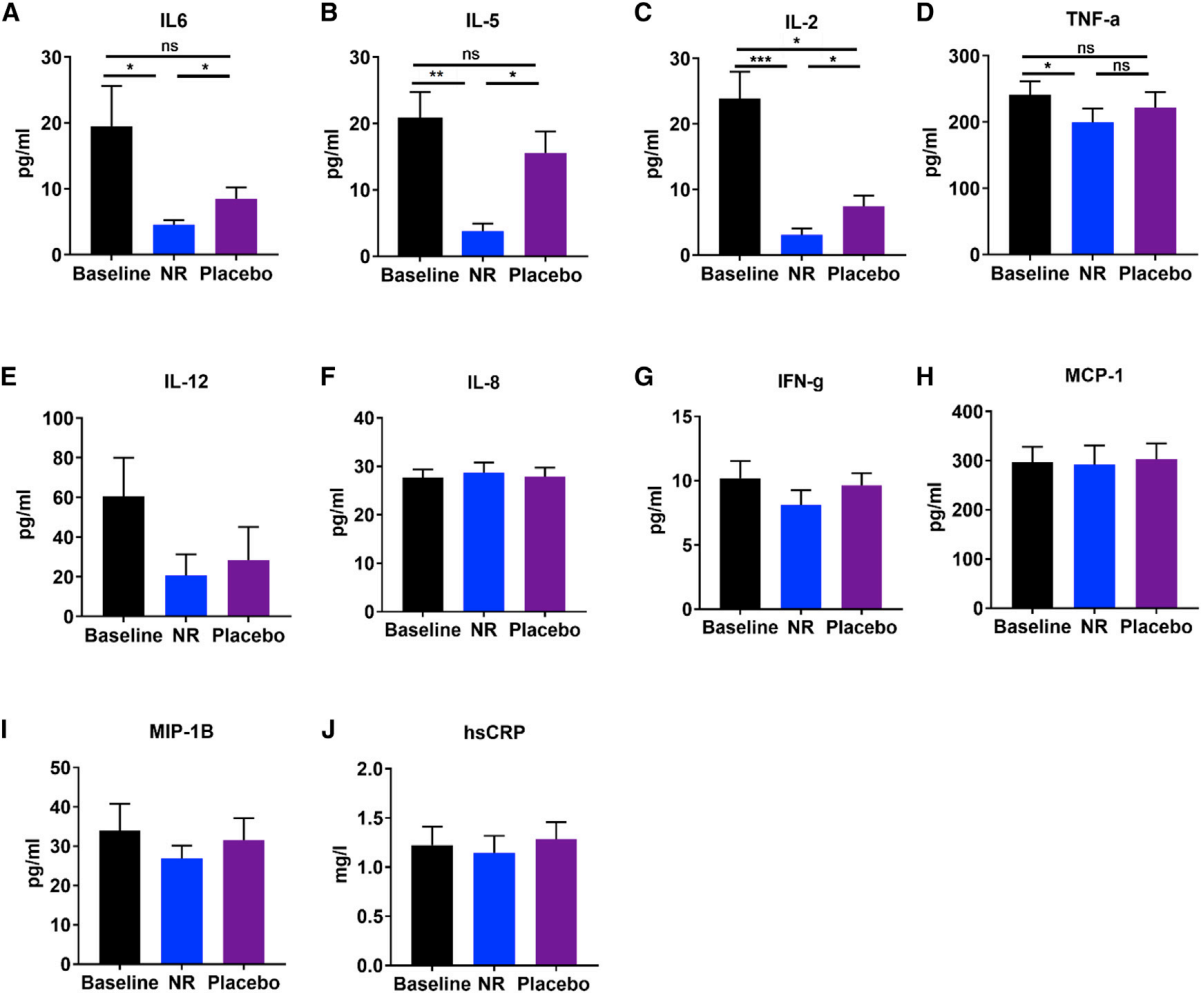
NR Supplementation Suppresses the Circulating Levels of Inflammatory Cytokines (A–J) Levels of serum inflammatory cytokines at baseline and after each of the NR and placebo phases, including (A) interleukin 6 (IL-6), (B) interleukin 5 (IL-5), (C) interleukin 2 (IL-2), (D) tumor necrosis factor alpha (TNF-α), (E) interleukin 12 (IL-12), (F) interleukin 8 (IL-8), (G) interferon-gamma (IFN-g), (H) monocyte chemoattractant protein-1 (MCP-1), (I) macrophage inflammatory protein-1 beta (MIP-1B), and (J) high-sensitivity C-reactive protein (hsCRP). Data are obtained from 12 participants at each phase and presented as mean ± SEM. Significance was set at p < 0.05 using paired t test. The absence of significance symbols indicates a lack of statistical significance.

Chronic inflammation appears to be a consistent feature of aging, even in apparently healthy individuals (Singh and Newman, 2011), and may contribute to age-related disturbances in metabolic homeostasis (Imai and Yoshino, 2013). We hypothesized that NR supplementation would reduce the levels of circulating inflammatory cytokines. We measured multiple inflammatory cytokines (see Method Details), 10 of which were within the assay detection range (Figure 6). NR significantly decreased the levels of the interleukins IL-6 (Figure 6A), IL-5 (Figure 6B), and IL-2 (Figure 6C) and tumor necrosis factor alpha (TNF-α) (Figure 6D), compared to baseline. We detected a statistically significant difference in the levels of IL-2 between baseline and the placebo (Figure 6C) and a lack of a difference in levels of TNF-α between NR and the placebo, despite a difference between NR and baseline (Figure 6D). This is seemingly due to the NR carry-over effect beyond the washout period, as evident by the period effect analysis (Figures S5A–S5D), confirming that the cohort randomized to the placebo first had no difference in IL-2 between baseline and the placebo (Figure S5C), and there was a difference in TNF-α between NR and the placebo (Figure S5D). No NR-mediated changes were detected in the serum levels of IL-12 (Figure 6E), IL-8 (Figure 6F), interferon-gamma (IFN-g) (Figure 6G), monocyte chemoattractant protein-1 (MCP-1) (Figure 6H), macrophage inflammatory protein-1 beta (MIP-1B) (Figure 6I), and high-sensitivity C-reactive protein (hsCRP) (Figure 6J). Thus, it will be interesting to further investigate depressed IL-6, IL-5, IL-2, and TNF-α as biomarkers and/or mediators of oral NR in rodent models and humans.

## Discussion

The NAD^+^ precursor NR has been studied extensively in animal and cell models. Its application *in vivo* has produced impressive results ameliorating metabolic dysfunction and muscle decline (Fang et al., 2017). No data exist on whether oral NR is available to human skeletal muscle, and data on tissue NAD^+^ content during aging are sparse, as are the consequences of NR supplementation in aged humans.

Using a robust clinical trial design, we show that 21 days of NR supplementation is safe and well tolerated in an aged male cohort and leads to an augmented NAD^+^ metabolome in whole blood, corroborating data recently reported by others (Martens et al., 2018, Trammell et al., 2016a). The median BMI in this trial is 26.6 kg/m^2^ (i.e., slightly overweight), but this is highly prevalent in aged populations (Winter et al., 2014) and may not indicate an unhealthy state (Porter Starr and Bales, 2015).

Experiments in genetic mouse models have shown that oral NR is available to cardiac (Diguet et al., 2018) and skeletal (Frederick et al., 2016) muscle, though it was also suggested that the benefit of extrahepatic NAD^+^ from oral NR is mediated by circulating NAM (Liu et al., 2018). Here, we show that oral NR increased human skeletal muscle NAAD, which was previously reported as a more sensitive marker of increased NAD^+^ metabolism than NAD^+^ per se (Trammell et al., 2016a), as well as MeNAM, Me-4-py, and Me-2-pywithout a rise in circulating NAM. Previous preclinical studies have established that oral NR is able to functionally restore muscle NAD^+^ despite a loss of NAM salvage (Frederick et al., 2016, Diguet et al., 2018). Although it is clear in rodent models that NR requires NR kinase activity in muscle (Ratajczak et al., 2016, Fletcher et al., 2017), further studies are required to understand NR dynamics in human muscle cells and tissues. Increased circulating levels of MeNAM and expression of its generating enzyme nicotinamide-N-methyltransferase (NNMT) have been associated with insulin resistance and type 2 diabetes (Kannt et al., 2015, Liu et al., 2015). However, the NR-mediated abundance of MeNAM did not alter glucose tolerance or substrate utilization in our study.

The levels of elevated NAM excretory products in skeletal muscle may be a result of pre-existing NAD^+^ sufficiency in this aged cohort and may explain the lack of effect on mitochondrial, physiological, and cardiometabolic parameters. A limited number of studies have reported minor age-related declines in NAD^+^ in human tissues (Massudi et al., 2012, Chaleckis et al., 2016, Zhou et al., 2016, Clement et al., 2018), but these data are non-conclusive. It is likely that a “second hit” arises during chronological aging that leads to tissue NAD^+^ decline and predisposes to age-related disease and frailty. This “second hit” may be conditions of metabolic stresses such as physical inactivity, chronic inflammation, or presence of a pre-existing cardiometabolic disease (e.g., obesity), and it may implicate downregulated NAMPT (Costford et al., 2010, Imai and Yoshino, 2013), depressed hepatic NADP(H) (Trammell et al., 2016b), and/or activation of CD38 (Camacho-Pereira et al., 2016, Covarrubias et al., 2019). Clearly, more human data are needed to delineate the relationship between aging and NAD^+^ metabolism.

We note a median hand-grip strength in our participants of 33.8 kg of force, consistent with muscle aging for men in their eighth decade and likely associated impairment in mitochondrial function. Our data suggest that 3 weeks of NR supplementation without concomitant muscle training is insufficient for increased strength.

NAD+ supplementation studies in rodents showed positive effects on muscle structural proteins (Frederick et al., 2016, Ryu et al., 2016, Zhang et al., 2016). It has been suggested that for muscle cell membranes, there is a capacity for NAD^+^-mediated ADP-ribosylation of integrin receptors that augment integrin and laminin binding and mobilize paxillin to bind adhesion complexes (Goody et al., 2010, Goody and Henry, 2018). Differential gene expression analysis may support this link and may highlight a potential role for NAD^+^ in the maintenance of skeletal muscle architecture, although this NR-induced transcriptomic signature appears to have no functional consequences at the protein level after the 21-day supplementation period. This observation may be important as we consider defective integrin and laminin structures such as in the context of muscular dystrophies (Mayer, 2003, McNally, 2012). Our data suggest a downregulation of gene sets associated with glycolysis and mitochondrial function, yet our measurements of mitochondrial respiration, citrate synthase activity, and mitochondrial copy number were unaltered. Again, expression levels of proteins involved in glycolytic and mitochondrial metabolism were unchanged with NR in this study. The downregulation of energy-generating processes may be reminiscent of mechanisms associated with calorie restriction (Hagopian et al., 2003, Ingram and Roth, 2011, Lin et al., 2015) or increased mitochondrial quality control, as has been observed in blood stem cells (Vannini et al., 2019), or it may suggest that NR can “tune” the expression of energy metabolism pathways to permit a more efficient and potentially stress-resilient mitochondrial environment. It will be interesting to further investigate those transcriptional changes in cell culture and animal models.

Some preclinical studies have reported that NR reduced macrophage infiltration in damaged muscle (Ryu et al., 2016, Zhang et al., 2016) and attenuated plasma TNF-α in models of fatty liver disease (Gariani et al., 2016). We show significant suppression of a number of circulating inflammatory cytokines. Studies are needed to explore the underlying mechanisms that mediate these NR-mediated anti-inflammatory effects. Of note, the expression of the NAD^+^-consuming enzyme CD38 increases in inflammatory cells with inflammation (Amici et al., 2018), as well as in the blood of aged humans (Polzonetti et al., 2012). Supplementing NAD^+^ in this context may be a mechanism mediating the NR-induced anti-inflammatory effects. Though chronic inflammation is a hallmark feature of aging (Singh and Newman, 2011), use of NR may yet find utility in other chronic inflammatory disorders, such as chronic obstructive pulmonary disease or rheumatoid arthritis, and is worthy of further investigation.

### Conclusions

We report that oral NR augments the aged human skeletal muscle NAD^+^ metabolome while inducing a transcriptional signature without affecting mitochondrial function or systemic cardiometabolic parameters. The targeted NAD^+^ metabolome analysis suggests pre-existing NAD^+^ sufficiency, despite hand-grip strength consistent with muscle aging. Our data may suggest that chronological age per se may not be a major factor in altering muscle and brain NAD^+^ metabolism, unlike aged laboratory mice. A limitation of this trial may be the number of participants or the duration of NR administration; however, the sample size was sufficient to detect NR-driven changes in the NAD^+^ metabolome, muscle transcriptional signature, and inflammatory profile. The transcriptional downregulation of mitochondrial gene sets also argues against the lack of a bioenergetic NR effect being due to the sample size. Further studies are needed to conceptualize some of the NR-mediated changes in this experimental medicine study.

Overall, these studies support that oral NR is available to human skeletal muscle, and they reveal anti-inflammatory NR properties, both of which may be beneficial in the context of aging, muscle, or inflammatory disease groups.

## STAR★Methods

### Key Resources Table

**Table:** 

REAGENT or RESOURCE	SOURCE	IDENTIFIER
**Antibodies**		

Custom made secondary antibodies (rabbit & mouse)	MRC Phosphorylation and Ubiquitylation unit, University of Dundee	N/A
α-Acetyl lysine	Cytoskeleton	Cat#AAC03
α-Alpha tubulin	Santa-Cruz	Cat#sc-8035
α-ANXA1	Sigma	Cat#HPA011272
α-Beta actin	Sigma	Cat#A5441
α-OXPHOS	Abcam	Cat#ab110413
α-PGK1	Abcam	Cat#ab199438
α-PGM1	Protein Tech	Cat#15161-1-AP
α-PKM1	Protein Tech	Cat#15821-1-AP

**Chemicals, Peptides, and Recombinant Proteins**		

^13^C labeled nucleotides, nucleosides	Trammell and Brenner, 2013	N/A
^18^O- nicotinamide	Kolodziejska-Huben et al., 2002	N/A
^18^O nicotinamide riboside	Yang et al., 2007	N/A
2-mercaptoethanol	VWR	Cat#441435C
Acetonitrile, Optima LC/MS	Fisher Scientific	Cat#A955-4
Adenosine 5′-triphosphate disodium salt hydrate	Sigma-Aldrich	Cat#A2383
Adenosine 5′diphosphate	Sigma-Aldrich	Cat#A5285
Adenosine diphosphate	Sigma-Aldrich	Cat#1905
Adenosine monophosphate	Sigma-Aldrich	Cat#A2252
ADPR	Sigma-Aldrich	Cat#A0752
Ammonium acetate	Sigma-Aldrich	Cat#238074
Antimycin A	Sigma-Aldrich	Cat#A8674
Benazmidine	Millipore	Cat#S7124222
Bovine serum albumin	Sigma	Cat#A2153
BSA, essentially fatty acid free	Sigma-Aldrich	Cat#A6003
Calcium carbonate	Sigma-Aldrich	Cat#C4830
Catalase from bovine liver	Sigma-Aldrich	Cat#C9322
Chemiluminescent HRP substrate	Millipore	Cat#WBKLS0500
Cytidine	Sigma-Aldrich	Cat#C122106
Cytochrome *c*	Sigma-Aldrich	Cat#C7752
d_3_, ^18^O methyl nicotinamide	Trammell et al., 2016a	N/A
d_4_-nicotinic acid	CDN isotopes	Cat#D-4368
Dithiotreitol	Sigma-Aldrich	Cat#D0632
DL-octanoyl carnitine-HCl	Tocris bioscience	Cat#605
Dnase I, Rnase-free	Thermo Scientific	Cat#EN0521
EDTA	Sigma	Cat#E1644
EGTA	Sigma-Aldrich	Cat#E4378
EGTA	Sigma	Cat#E4378
FCCP	Sigma-Aldrich	Cat#2920
Glucose reagent	Werfen Ltd	Cat#00018250740
Glutamic acid monosodium salt hydrate	Sigma-Aldrich	Cat#G1626
Glycerol reagent	Randox	Cat#GY105
Glycine	VWR	Cat#0167
HEPES	Fluka	Cat#BP310-1
Imidazole	Sigma-Aldrich	Cat#56750
Inosine	Sigma-Aldrich	Cat#I4125
Inosine monophosphate	Sigma-Aldrich	Cat#57510
Lactate Reagent	Randox	Cat#LC2389
Lactobionic acid	Sigma-Aldrich	Cat#153516
LDS sample buffer	Invitrogen	Cat#NP0008
Magnesium chloride hexahydrate	Sigma-Aldrich	Cat#M2670
Malic acid	Sigma-Aldrich	Cat#M1000
MES Free Acid Hydrate	Sigma-Aldrich	Cat#M8250
N1-methyl-2-pyridone-5-carboxamide	TLC Pharmaceutical Standards	Cat#N-0621
N1-methyl-4-pyridone-3-carboxamide	TLC Pharmaceutical Standards	Cat#N-0627
NAAD	Sigma-Aldrich	Cat#N4256
NAD	Sigma-Aldrich	Cat#N0632
NADP	Sigma-Aldrich	Cat#N5755
NAR	gift	N/A
N-d_3_ methyl −4 pyridone-3-carboxamide	TLC Pharmaceutical Standards	Cat#N-0628
NEFA reagent	Randox	Cat#FA115
Nicotinamide	Sigma-Aldrich	Cat#72340
nicotinamide N-oxide	Sigma-Aldrich	Cat#N3258
Nicotinamide riboside	ChromaDex	Cat#ASB-00014315
Nicotinic acid	Sigma-Aldrich	Cat#N4126
N-methyl nicotinamide	Cayman Chemical	Cat#16604
NMN	Sigma-Aldrich	Cat#N3501
Phosphocreatine disodium salt hydrate	Sigma-Aldrich	Cat#P7936
Potassium phosphate monobasic	Sigma-Aldrich	Cat#P9791
Protease inhibitor cocktail	Roche	Cat#11873580001
Rotenone	Sigma-Aldrich	Cat#R8875
Saponin from Quillaja bark	Sigma-Aldrich	Cat#S7900
SDS	ITW	Cat#A1112
Skimmed milk powder	Cell Signaling	Cat#9999
Sodium chloride	VWR	Cat#27800.360
Sodium fluoride	Alfa Aesar	Cat#7681-49-4
Sodium orthovanodate	Aldrich	Cat#450243
Sodium pyrophosphate	Sigma	Cat#221368
Sodium succinate dibasic hexahydrate	Sigma-Aldrich	Cat#S2378
Sucrose	Sigma-Aldrich	Cat#S9378
Sucrose	VWR	Cat#0335
Taurine	Sigma-Aldrich	Cat#T8691
TRI Reagent	Sigma-Aldrich	Cat#T9414
Triglyceride reagent	Werfen Ltd	Cat#00018258740
Tris-base	Fisher	Cat#BP152
Tris-HCL	Fisher	Cat#BP153
Triton X-100	Sigma	Cat#101634725
Tween-20	VWR	Cat#0777
U-^13^C_6_ glucose	Cambridge Isotope	Cat#CLM-1396-pk
Water, Optima LC/MS	Fisher Scientific	Cat#W6-4
β-Glycerophosphate	Sigma	Cat#G9422

**Critical Commercial Assays**		

Citrate synthase assay kit	Sigma-Aldrich	Cat#CS0720
Coomassie protein assay reagent	Thermo Fisher	Cat#1856209
Insulin assay	Mercodia	Cat#10-1113-01

**Deposited Data**		

Raw and processed data files for RNA sequencing	This paper	GEO: GSE133261

**Oligonucleotides**		

Please refer to Table S6	This paper	N/A

**Other**		

Precast gels	BioRad	Cat#5671084
Thermo hypercarb 2.1 × 100 mm column, 3μm	Fisher	Cat#35003-102130

### Lead Contact and Materials Availability

Further information and requests for resources and reagents should be directed to and will be fulfilled by the Lead Contact, Gareth Lavery (g.g.lavery@bham.ac.uk). This study did not generate new unique reagents.

### Experimental Model and Subject Details

#### Study conduct

The study was conducted between July 2016 and August 2017 at the National Institute for Health Research/Wellcome Trust Clinical Research Facility at the Queen Elizabeth Hospital Birmingham, UK. The Solihull NRES Committee gave ethical approval (REC reference number 16/WM/0159). All participants provided written informed consent. The study was registered on www.clinicaltrials.gov (Identifier: NCT02950441).The study was undertaken according to the principles of the Declaration of Helsinki and followed the Guidelines for Good Clinical Practice.

#### Study population

Aged volunteers were recruited from the Birmingham 1000 Elders group (https://www.birmingham.ac.uk/research/activity/mds/centres/healthy-ageing/elders.aspx). All participants fulfilled the inclusion criteria including: male sex, age 70 – 80 years, BMI 20 – 30 kg/m^2^, able to discontinue aspirin for 3 days prior to the muscle biopsy, and able to discontinue statins and vitamin D supplements for a week before the study and for the duration of the study. Exclusion criteria included: serious active medical conditions including inflammatory diseases or malignancies, significant past medical history including diabetes mellitus, ischemic heart disease, cerebrovascular disease, respiratory disease requiring medication, or epilepsy, blood pressure >160/100mmHg, or treatment with oral anti-coagulants.

#### Study design

Single center, double blind, placebo-controlled, and crossover study. Aim was to obtain complete assessments from 12 aged individuals. Participants attended for a screening visit (visit 1) when an informed written consent was obtained after ensuring they fulfil all inclusion criteria. For all subsequent study visits (2 to 5), the participants attended at 08:00 in a fasting state from the night before. Regarding the post-interventions visits (3 and 5), the participants took the last NR/placebo dose 14 h prior to the assessments.

#### Randomization and blinding

Participants were allocated to either NR or placebo. A randomization list was held by the clinical trials pharmacist at the clinical research facility. The study investigators, nurses, and participants were all blinded to the intervention allocation during the trial.

#### Intervention

NR was supplied as 250 mg capsules by the manufacturer (Niagen, ChromaDex, Irvine, CA). Participants received NR 500mg twice daily or matched placebo for 21 days with 21 days washout period between the NR and placebo periods (Figure S1).

#### Assessments

Assessments undertaken in each study visits are detailed in Figure S1.

### Method Details

#### Blood pressure

Blood pressure (Welch Allyn, USA) was measured at the start of each visit after an overnight fast. At the trial visits, participants rested for 15mins in a supine position before blood pressure was measured. An appropriately sized cuff was selected to encircle at least 80% of the arm and the same was used every visit for all participants, and on the same arm. Blood pressure was measured in triplicates and the mean was recorded.

#### Hand-held dynamometry

Peak absolute strength (kilograms) and relative handgrip strength (kilograms of force per kilogram of body weight) were measured in triplicate bilaterally using a dynamometer (Takei Instruments, Japan). The highest measurement values were included for analysis.

#### Muscle biopsies

Resting vastus lateralis muscle biopsies were obtained from 12 men by a single investigator (Y.SE) using a percutaneous Bergstrom technique as previously described (Bergstrom, 1975) under local anesthesia (1% lignocaine). The biopsy sample (100 – 150mg) was immediately dried on clean filter paper and approximately 10mg of tissue was cut and placed on ice cold BIOPS buffer for high-resolution respirometry (see below). The rest of the muscle tissue was immediately snap frozen in liquid nitrogen and stored at −80°C pending analysis.

#### Indirect calorimetry

Participants were allowed to rest for 60 mins after insertion of the arterial and venous catheters. Then they lay supine in a comfortable position while a transparent ventilated canopy was placed over their head. Plastic sheet attached to the hood was placed around the subjects to form a seal. The room temperature, barometric pressure, and humidity were measured by a hygrometer (Oregon Scientific). During the measurement period, participants remained supine, and breathed normally. Respiratory measurements, including resting oxygen consumption (VO_2_) and carbon dioxide production (VCO_2_) and respiratory exchange ratio (VCO_2_/VO_2_), using the mixing chamber mode of the metabolic cart (AEI MOXUS II Metabolic System). Measurements were collected at fasting and then every 30 min for 2 h following a 75 g oral glucose load. Measurements for each period lasted 15 mins so the first and last min could be discarded, and the mean value for the middle 5 min was recorded.

#### Arterio-venous difference technique

An arterial catheter was inserted into a radial artery and a retrograde cannula was inserted into in a deep antecubital vein draining a forearm muscle, on the opposite side of the arterial line. To prevent contamination of the muscle venous blood with the mixed blood from the hand, a wrist cuff was inflated to 200mmHg for 3 mins before sampling. Blood sampling was undertaken simultaneously from the arterial and venous sites at fasting and every 20mins following oral glucose load for 120 min.

#### Venous occlusive plethysmography

Forearm muscle blood flow was measured by venous occlusive plethysmography (Hokanson, USA) (Wythe et al., 2015) as previously described (Greenfield et al., 1963). Blood flow measurements were taken immediately after each blood sampling.

### NAD^+^ metabolomics

Muscle tissue was pulverised and approximately 10 mg was used for each of the acid (A) and basic (B) metabolite extraction. For each sample, internal standard mixtures for each of A and B were prepared. The extraction was undertaken using 0.2 mL of ice-cold LC-MS/MS grade methanol and kept on ice before adding 0.3ml of internal standard made in LC-MS grade water. Samples were sonicated in an acetone water bath (at −4°C) for 20 s, placed back on ice, and then incubated at 85°C with constant shaking at 1050 rpm for 5 min. Samples were then placed on ice for 5 min and centrifuged (16.1k x g, 10 min, 4°C). The supernatant was transferred to clean tubes and dried using a speed vacuum. The dried extract was re-suspended in 30 μl of either LC-MS grade water for acid extract or 10 mM ammonium acetate for alkaline extract and centrifuged (16.1k x g, 3 min, 4°C). The supernatant was carefully transferred to a Waters Polypropylene 0.3 mL plastic screw- top vial. The pellet was then dried using a speed vacuum, pellet was weighed, and later used to normalize data that were finally reported as pmol/mg.

Otherwise, muscle, blood, and urine metabolomics were undertaken as previously described (Trammell and Brenner, 2013, Trammell et al., 2016a).

#### Blood biochemical analysis

Blood was drawn from the arterial and venous catheters into heparinised blood tubes. Plasma was rapidly separated by centrifugation at 4°C and was then snap frozen. Plasma glucose, NEFA, and lactate concentrations were measured using commercially available kits on an ILAB 650 Clinical Chemistry Analyzer (Werfen Ltd, UK). Insulin was measured using commercially available assay as per the manufacturer’s instructions (Mercodia, Sweden). Homeostasis model assessment of insulin resistance (HOMA-IR) was calculated using the formula [fasting glucose (mmol/L) × fasting insulin (mU/L)/22.5].

Lipid profile, urea and electrolytes, and thyroid function tests were all measured on the Roche Modular Platforms (Roche, Switzerland). Full blood count was measured on a Beckman Coulter DxH analyzer (USA).

#### High-resolution respirometry on permeabilized muscle fibers

*Ex vivo* mitochondrial function was determined by measuring oxygen consumption polarographically using a two-chamber Oxygraph-2k (OROBOROS Instruments). Oxygen consumption reflects the first derivative of the oxygen concentration (nmol/ml) in time in the respiration chambers and is termed oxygen flux [pmol/(s^∗^mg)], corrected for wet weight muscle tissue (2–5 mg) introduced into the chamber. Measurements were undertaken according to a previously described protocol (Pesta and Gnaiger, 2012). Similar results were obtained if respiration rates were corrected for mitochondrial DNA (mtDNA) copy number or citrate synthase activity.

#### Mitochondrial density assessments

For citrate synthase activity, 5mg of snap frozen human muscle was used and the measurement was undertaken as previously described (Horscroft et al., 2015). Mitochondrial DNA (mtDNA) copy number was determined using quantitative real time PCR. mtDNA copy number was calculated from the ratio of NADH dehydrogenase subunit 1 (ND1) to lipoprotein lipase (LPL) (mtDNA/nuclear DNA) as previously described (Phielix et al., 2008).

#### RNA sequencing

RNA was extracted from frozen muscle tissue using Tri Reagent (Sigma-Aldrich) following manufacturer’s instructions. Sequencing libraries were prepared using RNA (RIN > 7) with the Lexogen Quantseq3 FWD kit. Libraries were sequenced using HiSeq2000 across 4 flowcells generating 75bp long single ended reads (average read depth of 6-10M/sample, which is higher than the 4M reads / sample required for analysis for this type of library). All samples were prepared and sequenced as a single pool. Trimmomatic software (v0.32) and bbduk.sh script (Bbmap suite) were used to trim the ILLUMINA adapters, polyA tails and low quality bases from reads. Trimmed reads were then uniquely aligned to the human genome (hg38) using STAR with default settings (v2.5.2b) and the Gencode (v28, Ensembl release 92) annotation as the reference for splice junctions. Mapped reads were quantified using HT-seq (v0.9.1) using Gencode (v28) genes (-intersection-nonempty flag). Differential gene expression was obtained using DEseq2 with paired baseline and treatment samples.

In this analysis we did not use a cutoff to remove lowly expressed genes. Inclusion of lowly expressed genes (at arbitrary cut-offs) had little bearing on our results (97.8% of differentially expressed genes at p < 0.05 were identical between no cutoff, and a cut-off of >3). Of note, volcano plot was drawn with a cut-off (> 3) in order to visualize the typical “V” shape using R. Differentially expressed genes between baseline (control) and NR treated samples at p value = < 0.05 were annotated using Biological processes (BP) gene sets with DAVID tool. We obtained similar results using gene annotation tool within Gene Set Enrichment Analysis (GSEA) suite (Subramanian et al., 2005, Liberzon et al., 2015) for gene sets from KEGG pathways and C5-Biological processes.

In addition, we have used GSEA analysis tool to interrogate specific gene sets against our pre-ranked expression data (Control versus NR treatment). GSEA calculates an Enrichment Score (ES) by scanning a ranked-ordered list of genes (according to significance of differential expression (-log10 p value), increasing a running-sum statistic when a gene is in the gene set and decreasing it when it is not. The top of this list (red) contains genes upregulated upon NR+ treatment while the bottom of the list (blue) represents downregulated genes. Each time a gene from the interrogated gene set (i.e., Glycolysis, mitochondria, TCA cycle) is found along the list, a vertical black bar is plotted (hit). If the “hits” accumulate at the bottom of the list, then this gene set is enriched in upregulated genes (and vice versa). If interrogated genes are distributed homogenously across the rank ordered list of genes, then that gene set is not enriched in any of the gene expression profiles (i.e., control gene sets of similar expression levels to interrogated gene sets). GSEA was used in pre-ranked mode with parameters -norm meandiv -nperm 1000 -scoring_scheme weighted. 10 gene sets of equal size and similar expression levels to the interrogated gene sets were generated using a custom pipeline in R (available upon request). We have interrogated the following gene sets: GO0048870; cell motility, GO0030029; actin filament based process, GO0022610; Biological cell adhesion, (also GO0007155 cell adhesion with similar results), M15112: Wong Mitochondria gene module, M3985: KEGG citrate cycle TCA cycle, merge of M15109: BIOCARTA Glycolysis pathway and M5113: REACTOME glycolysis.

#### Protein immunoblotting

Muscle biopsies were homogenized in ice-cold sucrose lysis buffer (50 mM Tris/HCl (pH 7.5), 250 mM Sucrose, 10mM Na-β-Glycerophosphate, 5mM Na-Pyrophosphate, 1mM Benazmidine, 1 mM EDTA, 1 mM EGTA, 1% Triton X-100, 1 mM Na3VO4, 50 mM NaF, 0.1% β-Mercaptoethanol, supplemented with protease inhibitor cocktail). Samples (40-100μg of protein extract) were loaded into 4%–15% Tris/Glycine precast gels (BioRad) prior to electrophoresis. Proteins were transferred onto PVDF membranes (Millipore) for 1h at 100V. A 5% skimmed milk solution made up with Tris-buffered saline Tween-20 (TBS-T, 0.137M NaCl, 0.02M Tris-base 7.5pH, 0.1% Tween-20) was used to block each membrane for 1h before being incubated overnight at 4°C with appropriate primary antibodies. Membranes were washed in TBS-T three times prior to incubation in horse radish peroxidase-conjugated secondary antibody at room temperature for 1h. Membranes were then washed in TBS-T prior to antibody detection via enhanced chemiluminescence horseradish peroxidase substrate detection kit (Millipore). Images were undertaken using a G:Box Chemi-XR5 (Syngene).

#### Inflammatory cytokines

We performed a multiplex cytokine bead assay using the Bio-Plex Pro Human Cytkine 17-plex panel analyzed with a flow-cytometry based Luminex 200 reader. The levels of IL-1b, IL-2, IL-4, IL-5, IL-6, IL-7, IL-8, IL-10, IL-12, IL-13, IL-17, G-CSF, GM-CSF, IFN-g, MCP-1, MIP-1b, and TNF-a were measured on the participants’ sera as per the manufacturer’s instructions. Only IL-2, IL-5, IL-6, IL-8, IL-12, IFN-g, TNF-a, MCP-1, and MIP-1b were within detection range. High sensitive CRP was measured using CRPHS: ACN 217 on COBAS 6000 analyzer (Roche, USA). All measurements were undertaken in duplicates.

### Quantification and Statistical Analysis

Sample size for this experimental medicine study was decided upon based on previous experience from studies using the same methodological design, whereby the proposed sample size was sufficient to detect significant differences at the 5% level. The analysis was based on data from all participants who were randomized, and completed all the study visits and assessments. Outcome data were reported as mean ± SEM (or median and quartiles where appropriate). In the NR supplementation study, comparisons of participants between placebo and NR supplementation phases were undertaken using paired t tests. In addition, further data analysis taking into account the period effect was undertaken, by grouping the participants into those who were randomized to NR first and second. This is to look for carryover effect across all analyses. Wherever relevant, area under the curve was calculated using the trapezoid method. Data were analyzed using IBM SPSS Statistics version 22 and GraphPad Prism version 7.0.

### Data and Code Availability

Raw read files and processed data files for RNA sequencing can be found at the NCBI Gene Expression Omnibus (GEO) database (GSE133261). Scripts and other bioinformatics pipelines used to analyze RNA sequencing data can be found at https://github.com/iakerman/Quantseq.

## Supplemental Information

Document S1. Figures S1–S5 and Tables S1 and S2

Table S3. NAD+ Metabolomics Data in Skeletal Muscle, Blood, and Urine after Each of the Baseline, Nicotinamide Riboside (NR), Placebo, and Washout Phases (N = 12), Related to Figure 1

Table S4. List of Genes Up- and Downregulated in Skeletal Muscle Biopsies upon NR Supplementation (N = 12), Related to Figure 2

Table S5. List of Genes in Gene Ontology Terms Related to Processes Up- and Downregulated in Skeletal Muscle Biopsies upon NR Supplementation (N = 12), Related to Figure 2

Table S6. List of Primers Used in the Molecular Analysis, Related to STAR Methods

Document S2. Article plus Supplemental Information
