# Supplementary material for: Nicotinamide Riboside Augments the Aged Human Skeletal Muscle NAD+ Metabolome and Induces Transcriptomic and Anti-inflammatory Signatures
Source: Cell Rep. 2019 Aug 13;28(7):1717–1728.e6. doi: 10.1016/j.celrep.2019.07.043 (PMC6702140; doi:10.1016/j.celrep.2019.07.043)
Supplement: Document S1. Figures S1–S5 and Tables S1 and S2 [file mmc1.pdf]

Cell Reports, Volume *28*

Supplemental Information

Nicotinamide Riboside Augments the Aged Human Skeletal Muscle NAD$^+$ Metabolome and Induces Transcriptomic and Anti-inflammatory Signatures

Yasir S. Elhassan, Katarina Kluckova, Rachel S. Fletcher, Mark S. Schmidt, Antje Garten, Craig L. Doig, David M. Cartwright, Lucy Oakey, Claire V. Burley, Ned Jenkinson, Martin Wilson, Samuel J.E. Lucas, Ildem Akerman, Alex Seabright, Yu-Chiang Lai, Daniel A. Tennant, Peter Nightingale, Gareth A. Wallis, Konstantinos N. Manolopoulos, Charles Brenner, Andrew Philp, and Gareth G. Lavery

SUPPLEMENTAL INFORMATION

Suppl. Table 1. Related to Figures 1 and 5. Cardiometabolic parameters at baseline and after nicotinamide riboside (NR) and placebo. Data are presented as median (1st quartile, 3rd quartile).

Parameter	Baseline	NR	Placebo
**Age (years)**	75 (72, 78)	-	-
**Weight (kg)**	82.6 (76.5, 90.1)	83 (76.3, 89.9)	82.6 (77.0, 89.6)
**BMI (kg/m^2)**	26.6 (25.0, 30.0)	26.9 (24.9, 29.5)	26.9 (25.1, 29.3)
**Systolic blood pressure (mmHg)**	139 (136, 154)	138 (130, 147)	134 (126, 155)
**Diastolic blood pressure (mmHg)**	86 (83, 93)	84 (79, 93)	81 (77, 90)
**Fasting glucose (mmol/L)**	5.88 (5.63, 6.31)	6.04 (5.81, 6.23)	5.85 (5.67, 6.30)
**Fasting insulin (mU/L)**	7.07 (7.27, 8.67)	6.27 (5.47, 7.07)	7.06 (5.47, 8.67)
**Cholesterol (mmol/L)**	4.8 (3.6, 5.2)	4.6 (3.9, 5.6)	4.6 (4.2, 5.3)
**HDL cholesterol (mmol/L)**	1.4 (1.2, 1.5)	1.3 (1.2, 1.5)	1.4 (1.1, 1.5)
**Triglycerides (mmol/L)**	1.0 (0.6, 1.2)	1.0 (0.8, 1.2)	1.0 (0.7, 1.2)

Suppl. Table 2. Related to Figure 1. Safety parameters at baseline and after nicotinamide riboside (NR) and placebo. Data presented as median (1st quartile, 3rd quartile). ALT, alanine aminotransferase; TSH, thyroid stimulating hormone; Free T4, free thyroxine.

Parameter	Baseline	NR	Placebo
**Haemoglobin (g/L)**	139 (129, 145)	133 (128, 139)	136 (131, 149)
**White cell count (x10^9/L)**	6.2 (5.5, 7.0)	6.2 (5.3, 6.9)	6 .2 (5.7, 7.1)
**Platelets (x10^9/L)**	187 (174, 233)	184 (169, 221)	203 (171, 231)
**Sodium (mmol/l)**	140 (139, 143)	139.5 (137, 142)	141 (137, 142)
**Potassium (mmol/l)**	4.2 (4.0, 4.3)	4.3 (4.1, 4.4)	4.2 (4.1, 4.5)
**Urea (mmol/L)**	6.0 (4.2, 6.5)	4.6 (4.0, 5.8)	5.1 (4.4, 6.0)
**Creatinine (µmol/L)**	74 (64, 78)	71 (67, 85)	74 (67, 79)
**Calcium (mmol/L)**	2.3 (2.2, 2.4)	2.3 (2.2, 2.4)	2.3 (2.2, 2.3)
**Albumin (g/L)**	43 (42, 44)	42 (40, 42)	42 (39, 43)
**Total protein (g/L)**	65 (63, 70)	65 (63, 67)	65 (62, 67)
**Alkaline phosphatase (u/L)**	56 (45, 62)	55 (46, 61)	52 (48, 62)
**Bilirubin (umol/L)**	7 (6, 10)	8 (6, 10)	8 (6, 9)
**ALT (u/L)**	17 (14, 17)	14 (12, 15)	16 (13, 19)
**TSH (mIU/L)**	1.7 (1.0, 2.7)	1.8 (1.3, 2.8)	1.6 (1.1, 2.5)
**Free T4 (pmol/L)**	14.0 (12.1, 15.1)	13.7 (13.4, 17.4)	14.8 (13.1, 17.5)

Outcome	Measure	Day							
		Day 0 (Visit 1) Enrolment	Day 1 (Visit 2) Baseline assessments and randomization		Day 22 (Visit 3)		Day 43 (Visit 4) Crossover		Day 64 (visit 5)
Safety	Medical history and examination*	•	•		•		•		•
	Adverse events and compliance		•		•		•		•
	Blood tests**		•		•		•		•
Study assessments	Blood pressure	•	•		•		•		•
	Fasting blood		•	Nicotinamide riboside 500 mg twice daily or placebo	•	Washout	•	Nicotinamide riboside 500 mg twice daily or placebo	•
	Hand-grip strength		•		•				•
	Muscle biopsy		•		•				•
	Glucose tolerance test		•		•				•
	Indirect calorimetry		•		•				•
	Muscle arterio-venous difference technique		•		•				•
	Venous occlusive plethysmography		•		•				•
	24 hour urine		•		•		•		•

Suppl. Figure 1. Schedule of study visits. Related to STAR methods.
Outline of the measures undertaken in each of the 5 study visits and the time interval between visits. *Body weight and height, systemic examination, and resting electrocardiogram. ** Full blood count, and renal, liver, and thyroid functions.

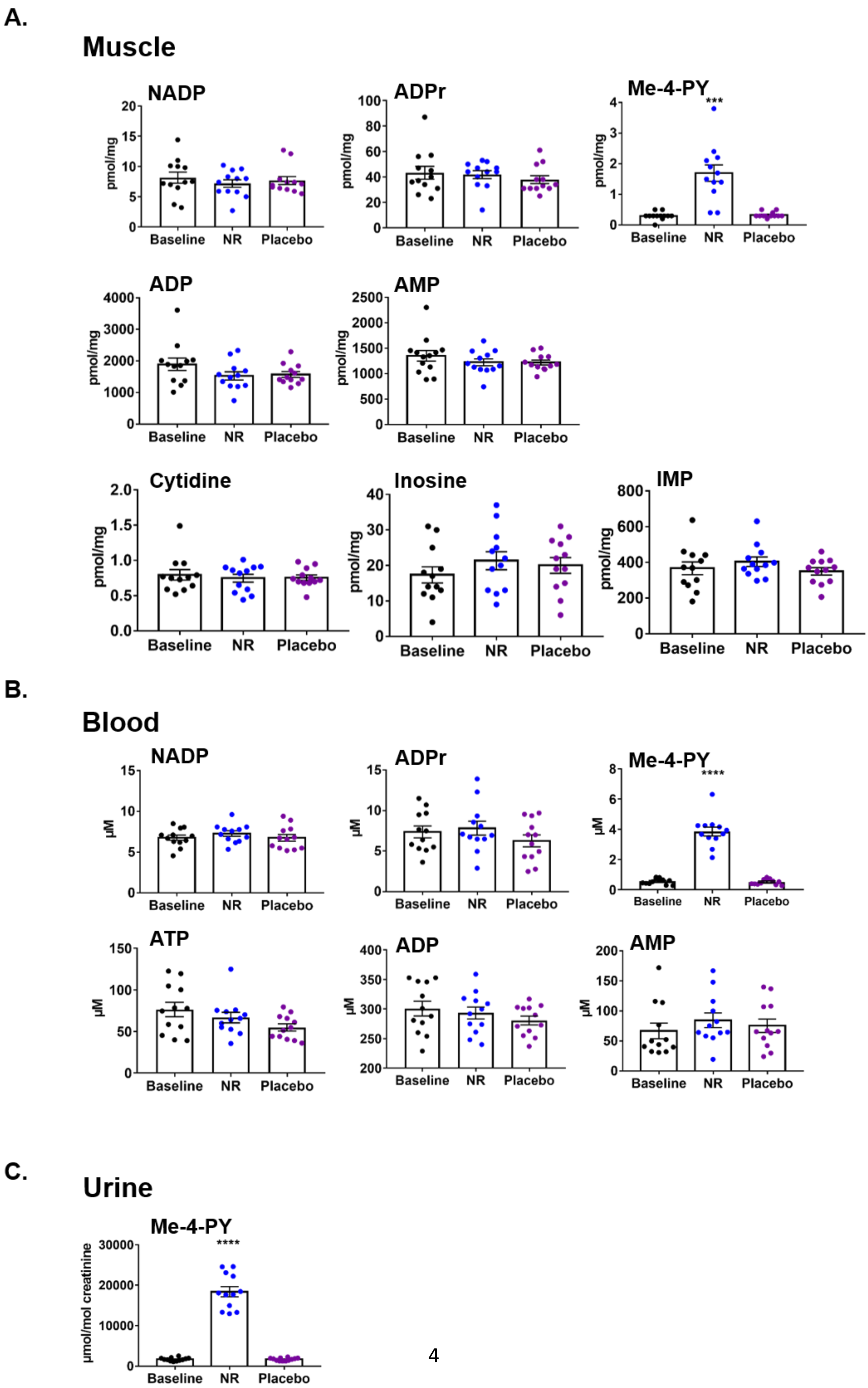
A.
Muscle
NADP
ADPr
Me-4-PY
***
ADP
AMP
Cytidine
Inosine
IMP
pmol/mg
Baseline
NR
Placebo
B.
Blood
NADP
ADPr
Me-4-PY
****
ATP
ADP
AMP
µM
Baseline
NR
Placebo
C.
Urine
Me-4-PY
****
µmol/mol creatinine
Baseline
NR
Placebo

Suppl. Figure 2. NAD$^+$ metabolomics in skeletal muscle, whole blood and urine. Related to Figure 1.

Remainder of LC-MS/MS NAD$^+$ metabolomics in (A) skeletal muscle, (B) whole blood, and (C) urine, which were not shown in Figure 1. NADP, nicotinamide adenine dinucleotide phosphate; ADPr, adenosine diphosphate ribose, Me-4-py, N1-Methyl-4-pyridone-5-carboxamide; ATP, adenosine triphosphate; ADP, adenosine diphosphate; AMP, adenosine monophosphate. Data are obtained from 12 participants at each phase and presented as mean ± SEM. Significance was set at $p < 0.05$ using paired t-test. The absence of significance symbols indicates lack of statistical significance.

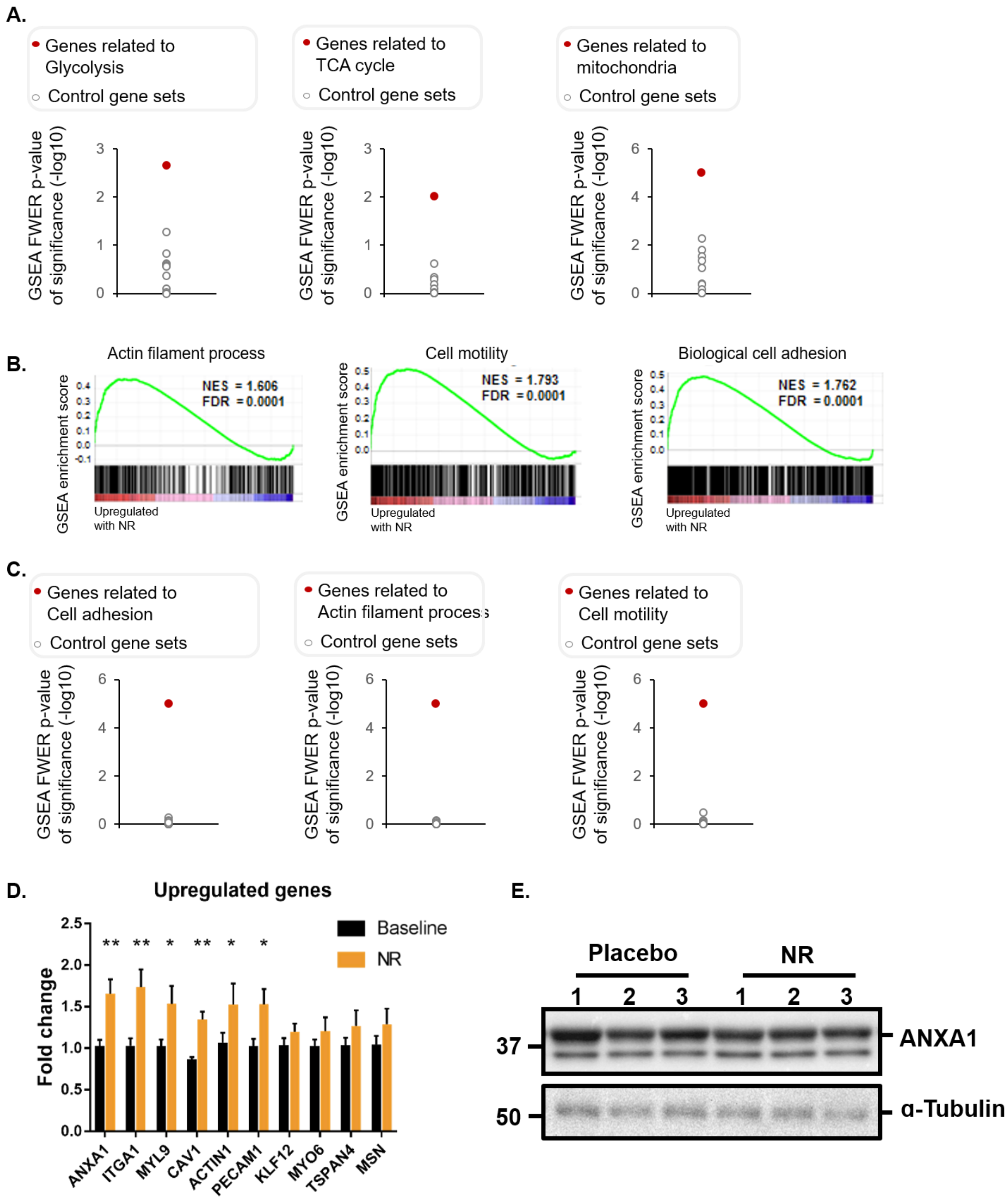

Suppl. Figure 3. Skeletal muscle RNA sequencing. Related to Figure 2.
(A) Bar plots show GSEA p-value of significance (-Log10) for enrichment of genes belonging to the pathways gylcolysis, TCA cycle and mitochondria for downregulated targets of NR supplementation (in red). The same analysis on 10 gene sets of equal size and expression level do not reveal enrichment amongst downregulated targets of NR (in grey). (B) Gene set enrichment analysis (GSEA) suggests that genes belonging to the gene sets

“Actin filament process”, “Cell motility”, and “Biological cell adhesion” are upregulated upon NR supplementation. Normalized enrichment score (NES) and nominal p-value is presented on the top left corner of the graph.

(C) Bar plots show GSEA p-value of significance (-Log10) for enrichment of genes belonging to the pathways “Actin filament process”, “Cell motility”, and “Biological cell adhesion” for upregulated targets of NR supplementation (in red). The same analysis on 10 gene sets of equal size and expression level do not reveal enrichment amongst upregulated targets of NR (ingrey).

(D) Quantitative PCR analysis of a select panel of upregulated genes identified through differential gene expression analysis. GAPDH was used as housekeeping gene. Error bars represent SEM (n=12).

(E) Quantification of Annexin A1 (ANXA1) protein using immunoblotting assay. Tubulin was used as a loading control.

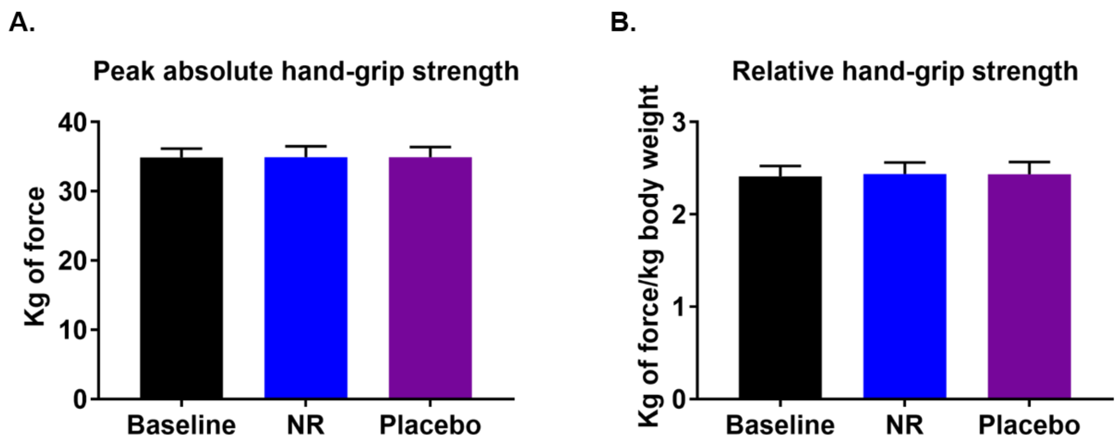

Suppl. Figure 4. Hand-grip strength. Related to Figure3.
(A) Peak hand-grip strength at baseline and after each of the nicotinamide riboside (NR) and placebo phases. (B) Similar to (A) but data presented relative to body weight. Data are obtained from 12 participants at each phase and presented as mean ± SEM. Significance was set at $p < 0.05$ using paired t-test. The absence of significance symbols indicates lack of statistical significance.

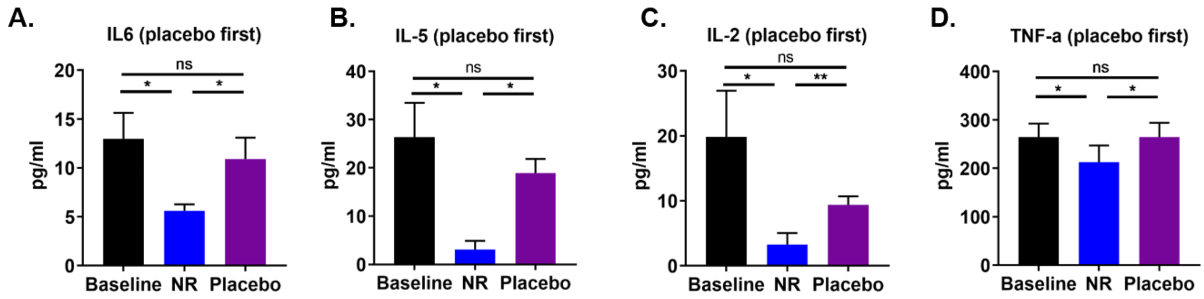

Suppl. Figure 5. Inflammatory cytokines period effect. Related to Figure 6.
Levels of serum inflammatory cytokines at baseline and after each of the nicotinamide riboside (NR) and placebo phases, including: (A) interleukin 6 (IL-6), (B) interleukin 5 (IL-5), (C) interleukin 2 (IL-2), and (D) tumor necrosis factor-alpha (TNF-a). Here period effect analysis is shown and each panel is produced from only the 6 subjects that were randomized to placebo first as a demonstration of the NR carryover effect, evident in the cases IL-2 (C) and TNF-a (D). Data are presented as mean ± SEM. Significance was set at $p < 0.05$ using paired t-test.
